# Phosphorus Shapes Soil Microbial Community Composition and Network Properties During Grassland Expansion Into Shrubs in Tibetan Dry Valleys

**DOI:** 10.3389/fpls.2022.848691

**Published:** 2022-03-23

**Authors:** Hanchang Zhou, Anzhou Ma, Xiaorong Zhou, Xianke Chen, Jiejie Zhang, Qinwei Zhang, Xiangning Qi, Guohua Liu, Guoqiang Zhuang

**Affiliations:** ^1^Research Centre for Eco-Environmental Sciences, Chinese Academy of Sciences, Beijing, China; ^2^College of Resources and Environment, University of Chinese Academy of Sciences, Beijing, China

**Keywords:** phosphorus, composition, network, Tibetan dry valleys, soil microbial community

## Abstract

Alpine ecosystem stability and biodiversity of the Tibetan plateau are facing threat from dry valley vegetation uplift expansion, a process which is highly connected to variations in the soil microbial community and soil nutrients. However, the variation of microbial community properties and their relationship to soil nutrients have scarcely been explored in Tibetan dry valleys, which is a gap that hampers understanding the dry valley ecosystem’s response to vegetation change. In this study, we sampled grasslands (G), a grass-shrub transition area (T), and shrublands (S) along an uplift expansion gradient and investigated the link between microbial community properties and soil nutrients. The results showed that shrub degradation by grass expansion in Tibetan dry valley was accompanied by increasing relative phosphorus (P) limitation, which was the main driver for bacterial and fungal composition variation as it offered highest total effect on PC1 (0.38 and 0.63, respectively). Total phosphorus (TP) was in the center module of bacterial and fungal network under shrub soil and even acted as key nodes in fungal networks. During the replacement by grass, TP was gradually marginalized from both bacterial and fungal center network module and finally disappeared in networks, with ammonia and nitrate gradually appearing in the bacterial network. However, TC and total nitrogen (TN) were always present in the center modules of both fungal and bacterial network. These support that a TP variation-induced compositional and network functional shift in the microbial community was a potential reason for vegetation uplift expansion in Tibetan dry valley. This study highlighted the effect of TP on microbial community properties during dry valley vegetation uplift expansion and offered basic information on Tibetan alpine dry valley ecosystem’s response to climate change.

## Introduction

Dry valleys are important components of the Tibetan plateau alpine ecosystem (Li, 2001; Zhao et al., 2015). Compared with other alpine ecosystems, the main vegetation of Tibetan dry valley is grass and shrub (Li, 2001; Zhao et al., 2015), in which the relatively lower biodiversity leads dry valley vegetation sensitive to climate change (Li, 2001; Zhao et al., 2015). Since the 1930s, the dry valley vegetation in Tibet plateau has shown an uplift expansion of over 300 m (Li, 2001; Zhao et al., 2015), with continued invasion and replacement of other alpine ecosystems, severely threatening the stability and biodiversity of the entire Tibetan plateau (Liu and Zhu, 2016). Unfortunately, very little research has focused on dry valley ecosystems as compared with other alpine ecosystems, especially on the aspect of lower grass uplift expansion into higher shrub (Qiong et al., 2010; Liu and Zhu, 2016), which deeply hampers the comprehensive understanding to the reasons and mechanisms of dry valley ecosystem’s response to current vegetation change, as well for local biodiversity conservation (Lindenmayer and Fischer, 2007; Harris et al., 2018).

The vegetation expansion between shrub and grass is always accompanied by the change in phosphorus (P) and the chemical stoichiometry (Yong et al., 2018; Gao et al., 2021), as shrub commonly had higher P content in biomass and higher P demand than grass (Yang et al., 2016; Carrillo et al., 2017). In nature terrestrial ecosystem, P originates from slow weathering of parental material and atmosphere ash deposition, cycles in different nutrient pools, and relocates in soil profiles (Walker and Syers, 1976; Canfield et al., 2005). To meet the higher P demand, shrubs uptake deep soil layer P into biomass *via* the root and release P as litters, which lead P accumulate in topsoil (Yong et al., 2018; Gao et al., 2021). Then, rain water moves the topsoil P downward into deeper active root area, and the P can be absorbed by shrub again (Walker and Syers, 1976; Canfield et al., 2005). Thus, a rapid, high-mobile P fraction-containing cycling was formed to benefit shrub dominance (Dinh et al., 2017).

The soil microbial community plays an irreplaceable role in accelerating the P cycle and improving high-mobile P fraction (Walker and Syers, 1976; Canfield et al., 2005). Microorganisms release extracellular enzymes and organic compounds to dissolute P-containing minerals and to mineralize litter organic P, thus enhancing the soil phosphate content (Walker and Syers, 1976; Canfield et al., 2005). Microorganisms also act as a temporally available P sink through microbial uptake and anabolism (Turner et al., 2013; Dinh et al., 2017). As a high richness system comprised fast reproductive potential members, the composition and function of the microbial community are sensitive to moisture and temperature variation, which makes it a contributor to soil nutrient content variation under climate change (Curtis, 2006; Singh et al., 2010; de Vries and Ashley, 2013). For example, frequent dry-wet cycling under an increasing irregular precipitation pattern stimulated microbial nitrification and denitrification, accelerating soil nitrogen loss as N_2_O and N_2_ (Kuypers et al., 2018). Rising temperature directly stimulates microbial community respiration and reduces soil carbon storage (Dong and Somero, 2009; Crowther et al., 2016). Thus, the P -related microbial community’s composition and functional changes are potential reasons for the grass-shrub expansion happened in Tibetan alpine dry valley. However, till present, there is still scarce research that had elucidated the microbial community change under such situation.

To fill the gap mentioned above, topsoil along the grass-shrub expansion gradient was sampled from a typical dry valley ecosystem located in the eastern edge of Tibetan plateau, and the soil microbial community’s property variations were explored. We hypothesized that (1) shrub degradation caused by grass uplift expansion is accompanied by decreasing topsoil P; (2) the P change is correlated with soil microbial composition; and (3) the microbial network in shrub soil tends to focus more on P.

## Materials and Methods

### Site Description and Sampling

Photos of the sampling sites are shown in Supplementary Figure 1, from low elevation to high elevation; grassland (G) (28°40′58.05″N, 101°12′8.56″E, 2,212 m), transition area of grass and shrubs (T) (28°40′59.38″N, 101°12′1.91″E, 2,340 m), and shrubland (S) (28°41′0.18″N, 101°11′55.16″E, 2,450 m) were sampled, respectively. The sites were located on an eastern facing slope above the Yalong River, near Bowo village (Sichuan Province); the slope degree ranged from ∼10°–45°. The mean annual temperature was about 11.59°C, and the mean annual precipitation was about 820 mm; climatic data were extracted from the “WorldClim-Global Climate Data”^1^. The soil samples were collected in August 2020. Ten composite samples (minimum of 10 m between samples) were collected uniformly at each site from 1 m × 1 m square to cover the within-site variability. For each composite sample, approximately 500 g top soil (0–10 cm) of each sampling spots were cored from 4 corners and the center of the square and thoroughly mixed. After roughly removing plant debris, small rocks, and animal bodies, ∼500 g was packed and stored in an ice box. The samples were transported at 4°C to the laboratory as soon as possible, after which, all samples were passed through the 2 mm sieves and divided into two parts, with one portion stored at −80°C for molecular analyses and the other portion stored at 4°C for the analysis of soil physicochemical property.

### Basic Soil Abiotic Properties Measurement

Soil pH was measured using a pH meter (FE20-FiveEasyTM pH, MettlerToledo, Germany) after shaking a soil water (1:2.5 w/v) suspension for 30 min (Guo et al., 2017; Zhou et al., 2022). Soil moisture was measured gravimetrically (Guo et al., 2017; Zhou et al., 2022). Total carbon (TC) and total nitrogen (TN) contents were measured using an elemental analyzer (VarioMAX, Elementar, Germany) (Guo et al., 2017; Zhou et al., 2022). Ammonium (NH_4_^+^-N) and nitrate (NO_3_^–^-N) were extracted at a ratio of 10 g fresh soil to 50 ml 2 mol L^–1^ KCl. After shaking under 180 r min^–1^ for 1 h, NH_4_^+^-N and NO_3_^–^-N contents in the filtered extracts were analyzed using a continuous flow analytical system (San^++^ System, Skalar, Holland) (Guo et al., 2017; Zhou et al., 2022). Total phosphorus (TP) content was measured using the alkali fusion-Mo-Sb antispectrophotometric method (Guo et al., 2017; Zhou et al., 2022).

### DNA Extraction and Sequencing

Soil DNA was extracted according to the manufacturer’s protocol using the FastDNA™ SPIN kit (MP Biomedicals); the DNA concentrations and quality were checked using a Nano-100 NanoDrop spectrophotometer. Primers for 16S rRNA gene amplification targeted the V3-V4 hypervariable region and included 338F 5′-ACTCCTACGGGAGGCAGCA-3′ and 806R 5′-GGACTACHVGGGTWTCTAAT-3′ (Mori et al., 2014). The primers for ITS2 region amplification included 3F 5′-GCATCGATGAAGAACGCAGC-3′ and 4R 5′-TCCTCCGCTTATTGATATGC-3′ (Toju et al., 2012). PCR amplification was performed in 50.0 μl reaction systems containing 25.0 μl of Premix Taq DNA polymerase, 0.5 μl of the forward primer (20 mM), 0.5 μl of the reverse primer (20 mM), 23.0 μl of double-distilled water (ddH_2_O), and 1.0 μl of the DNA template (20 ng total DNA) (Liu et al., 2021). Amplicons were pooled in equimolar ratios and paired-end sequenced on the Illumina Nova6000 platform (Majorbio Company, Shanghai). Majorbio Cloud Platform^2^ was used to conduct all of the sequence bioinformatic scripts; sequences were merged with a minimum overlap length of 20 bp into full-length sequences by FLASH after removing barcodes and primers (Magoč and Salzberg, 2011; Kai et al., 2017). UPARSE was used to remove chimeras and cluster operational taxonomic units (OTUs) defined at the 97% similarity level (Edgar, 2013). The bacterial OTUs were annotated according to the Sliva-138 database, while the fungal OTUs were annotated according to the UNITE-8.0 database. Reads number of 26,349 (bacterial) and 101,210 (fungal) sequences were randomly selected from each sample to form an evenly resampled OTU table for further analysis. The raw sequences were uploaded onto NCBI, and the project numbers are listed in Supplementary Table 1.

### Data Analysis

Network analyses were conducted according to the molecular ecological network analysis pipeline (MENA^3^) protocols. The top 1,000 relative abundance bacterial OTUs (BOTUs) and all fungal OTUs (FOTUs) were selected for bacterial and fungal network construction, respectively, as they covered over 90% of the total reads and appeared in every sample, which meant they could provide meaningful information for network construction. The construction parameters for the bacterial network were set as majority = 10, missing fill = fill paired (0.0100), logarithm = y, and similarity = pcc. The construction parameters for the fungal network were set as majority = 8, missing fill = fill paired (0.0100), logarithm = y, and similarity = pcc. The threshold values ranging from 0.01 to 0.99 over 0.01 intervals were applied to the Pearson correlation matrix for all three sites to scan for plausible network models, and cutoff threshold values of 0.92 ± 0.01 for bacterial network and 0.75 for fungal network were used to maintain a similar interaction strength comparison among the three different elevation sites (*p* > 0.05). Nodes with Pi (among-module connectivity) > 0.625 were divided into connectors, nodes of Zi (within-module connectivity) > 2.5 were divided into module hubs, and nodes of both Pi > 0.625 and Zi > 2.5 were divided into network hubs or were peripherals (Deng and Zhou, 2015; Xie et al., 2020). The constructed networks were visualized using the Cytoscape 3.3.0 software. Nearly, all parameters between the empirical networks and their corresponding random networks had significant differences, which indicated a plausible network construction and analysis (Supplementary Table 2). The statistically significant differences in basic soil properties and diversity indices among sites were measured using one-way ANOVA (Tamhane’s T2 test and Tukey’s HSD according to normality and homoscedasticity of data). Calculations of richness, Shannon index, and principal coordinates analysis (PCoA) based on Bray-Curtis distances at the OTU level were conducted with the online platform https://cloud.majorbio.com using R base (Edwards et al., 2015). The coordinates of the main axes, PC1 and PC2, from the PCoA analysis results for bacterial and fungal community were selected as community structure indicators to conduct further analysis, and the analysis of similarities (ANOISM) test was adopted to check the composition differences among the three sites; the *p* < 0.001 indicated significant compositional differences among sites (He and Xie, 2020). The scanning electron microscopic (SEM) analysis was conducted using the Amos software (Kline et al., 2011). Factor extractions were conducted using a Pearson correlation matrix in the SPSS software suite (version 24) (Ramette, 2007; Xie et al., 2020). In SEM, P limitation was extracted from C:P and N:P (molar ratio), two common indicators for measuring relative P limitation. The previous research has reported that global soil C:N:P stoichiometry averaged around 186:13:1 and soil microbial C:N:P stoichiometry averaged around 60:7:1 (Cleveland and Liptzin, 2007). The overly high C:P and N:P values are signs of soil P limitation, and the increasing C:P and N:P values indicate the enhanced relative P limitation to carbon and nitrogen (Cleveland and Liptzin, 2007). The variation from C:P and N:P was 0.96, and in total, they offered 91.42% explanation power. N resources were extracted from nitrate and ammonia; the variation from them was 0.84, and they offered 71.20% explanation power in total. Diversity was represented by bacterial or fungal Shannon indices in the corresponding SEMs. Composition was represented by bacterial or fungal PC1 of the PCoA analysis results in the corresponding SEM.

## Results

### Basic Abiotic and Biotic Properties Variation

**FIGURE 1:**
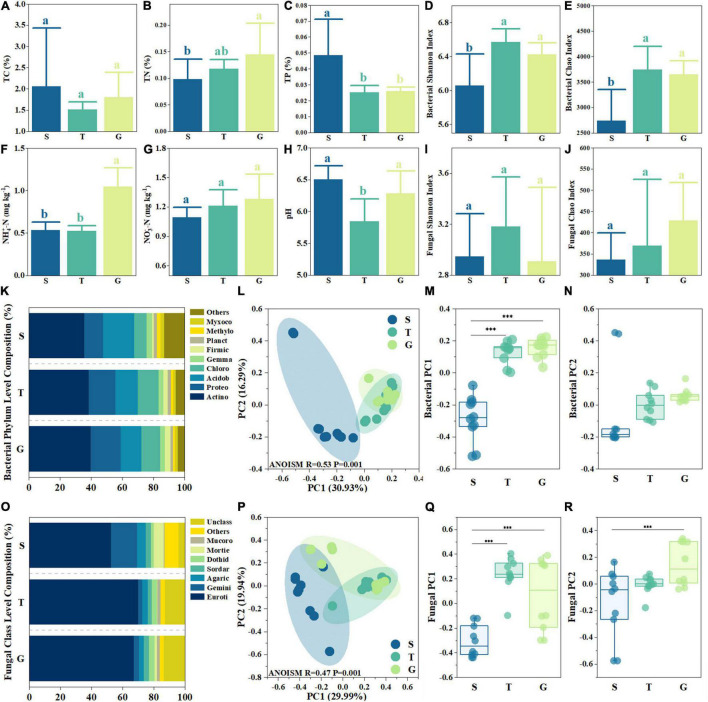
Basic abiotic and biotic site properties. Shrubland (S), transitional (T), and Grass (G) in each sub-figures indicate shrub, transition, and grassland. Subfigures **(A–J)** depict soil total carbon (TC), total nitrogen (TN), total phosphorus (TP), ammonia content, nitrate content, pH, bacterial and fungal community Shannon index, and Chao index. The bars in the histogram indicate the average, and the error bars indicate standard deviation (*n* = 10). The different letters inside subfigures **(A–J)** indicate significant differences between groups (*p* < 0.05, one-way ANOVA Tukey’s test). **(K,O)** depict the bacterial phylum composition and fungal class composition, respectively, with six letter abbreviations adopted for bacterial phylum and fungal class names. **(L,P)** depict principal coordinates analysis (PcoA) analysis for bacterial community and fungal community at the OUT level, respectively. The ^***^ in subfigures **(M,N,Q,R)** indicate significant differences between groups (*p* < 0.05, one-way ANOVA Tamhaney’s T2 test).

During replacement of shrubland by grassland, the relative P limitation (C:P and N:P) gradually increased due to the significant change in TP and TN. TP in S was about 0.8 times higher than T and G (*p* < 0.05). On the contrary, TN showed an increasing trend from S (0.10% ± 0.04%) to T (0.12% ± 0.02%) and G (0.15% ± 0.06%), and S was significantly lower than G (*p* < 0.05). Thus, N:P ratios increased from 4.76 ± 0.91 (S) to 10.55 ± 1.85 (T) and 12.11 ± 4.09 (G), which indicated a decreasing relative N limitation and increasing P limitation. Compared with TN, TC showed no significant differences among the S, G, and T stages (*p* > 0.05), which were lowest in T (1.52% ± 0.18%), which was about 75% of the highest S (Figure 1A). However, due to the decreasing of TP, C:P also increased from 111.77 ± 31.67 (S) to finally 176.99 ± 48.18 (G), which indicated increasing relative P limitation and decreasing relative C limitation. Similar to TN, ammonia also showed an increasing trend from S to G. The highest ammonia nitrogen content was observed in G (1.05 ± 0.23 mg kg^–1^), which was about two times that of S and T (*p* < 0.05) (Figure 1F). Nitrate nitrogen increased from 1.10 ± 0.10 mg kg^–1^ (S) to 1.28 ± 0.25 mg kg^–1^ (G), although no significant differences were found among the three sites (Figure 1G). These indicated that G possessed a higher labile nitrogen content pool rather than S. The pH in T was 5.84 ± 0.35, which was significantly lower (*p* < 0.05) than S (6.50 ± 0.21) and G (6.28 ± 0.35) (Figure 1H).

The bacterial Shannon and Chao indices increased significantly (*p* < 0.05) from S (6.06 ± 0.00 and 2,739 ± 616, respectively) to T (6.57 ± 0.16 and 3,743 ± 453, respectively) and then displayed a slight but non-significant decrease (*p* > 0.05) (Figures 1D,E). The fungal Shannon index was highest in T (3.18 ± 0.39) (Figure 1I), while non-significant differences were found among the three stages (*p* > 0.05). The fungal Chao index also displayed a non-significant (*p* > 0.05) increase from S (336 ± 63) to G (429 ± 89) (Figure 1J).

From S to G, most bacterial phyla showed linear changes in relative abundance (RA) (Figure 1K). For example, the RA of *Proteobacteria* in G (19.19%) was 0.6 times higher than S (11.98%), the RA of *Chloroflexi* also increased from 8.17% (S) to 12.17% (G) and 13.44% (T). Instead of the increasing trend, the RA of Acidobacteriota decreased from 20.03% (S) to 13.36% (G), and RA of “Others,” a group composed by rare species (less than 2% RA), was 13.14% in S and only 4.41% in G, decreased even nearly 70%. However, the most abundant phylum, Actinobacteriota, shared similar RA among three sites, which only slightly increased from S (36.65%) to G (39.77%). The most dominant fungal Class was Eurotiomycetes (Figure 1O), for which RA increased from 52.59% (S) to 70.01% (T) and 67.17% (G). The second most dominant fungal Class was Geminibasidiomycetes (RA 16.62% in S), whose RA shrank to 2.53% (T) and 3.30% (G). However, the RA of group “Unclassified” was 4.02% in S but 13.41% in G, while other groups also showed several differences among the three stages, like Dothideomycetes, whose RA at G and T was about twice that of S (1.61%).

The PCoA analysis of bacterial and fungal community composition at the OUT level found that there were significant differences in the community structure among the three stages (ANOISM *p* < 0.001). PC1 and PC2 of the bacterial community were 30.93 and 16.29%, respectively, while PC1 and PC2 of the fungal community were 29.99 and 19.94%, respectively (Figures 1L,P). Significant differences were found between bacterial PC1 of S and T and of S and G (*p* < 0.05), but not between T and G (*p* > 0.05) (Figure 1M). Fungal PC1 showed a similar trend (Figure 1Q). However, there were no significant differences among bacterial PC2 of the three stages (*p* > 0.05) (Figure 1N), while fungal PC2 of S and G was significantly different (*p* < 0.05) (Figure 1R). These results indicated that the variation in microbial composition during vegetation uplift expansion mainly occurred during the first stage of expansion (S to T) rather than the final stage of expansion (T to G).

### Structure Equation Modeling on Microbial Composition

**FIGURE 2:**
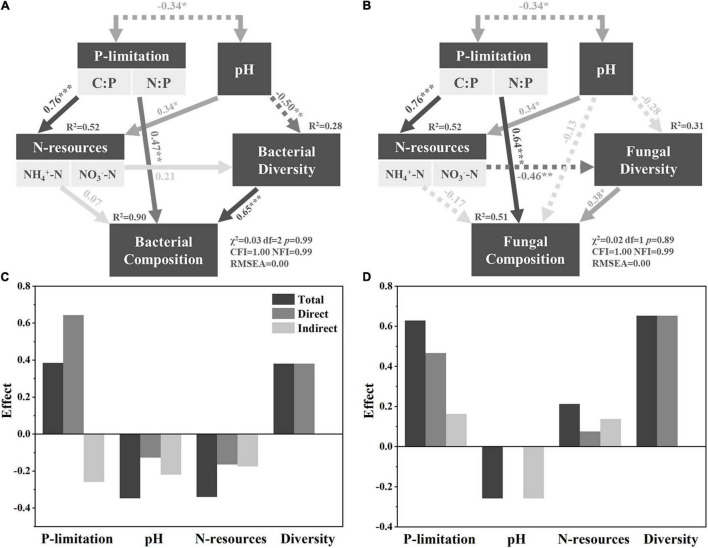
SEM resolving bacterial composition and fungal composition. Panels **(A,B)** are detailed SEMs for bacterial and fungal composition, respectively, while panels **(C,D)** show the effects of different factors on composition accordingly. Solid and dashed lines indicate positive and negative correlations, respectively. *, **, and *** indicate significant correlated under *p* < 0.05, *p* < 0.01, and *p* < 0.001, respectively.

The null model construction is depicted in detail in Supplementary Figure 2. The SEM showed that P limitation offered the highest total effect (0.38), direct effect (0.64), and indirect effect (−0.26) on fungal composition. Although the total effect was only 0.04 higher than pH and N resources, direct effect was almost four times and three times of pH and N resources, respectively. Indirect effect was 17.8% higher than pH and 48% higher than N resources, respectively (Figure 2C). The total effect of P limitation on bacterial composition was as high as 0.63, which was about 2.4 times that of pH (total effect, −0.26) and three times of N resources (0.21). Its direct effect was also as high as 0.47, while pH was 0.02 and N resources was 0.22 (Figure 2D). These results indicated that P limitation was the greatest contributor to microbial composition during vegetation variation.

### Network Analysis on Microbial Community

**FIGURE 3:**
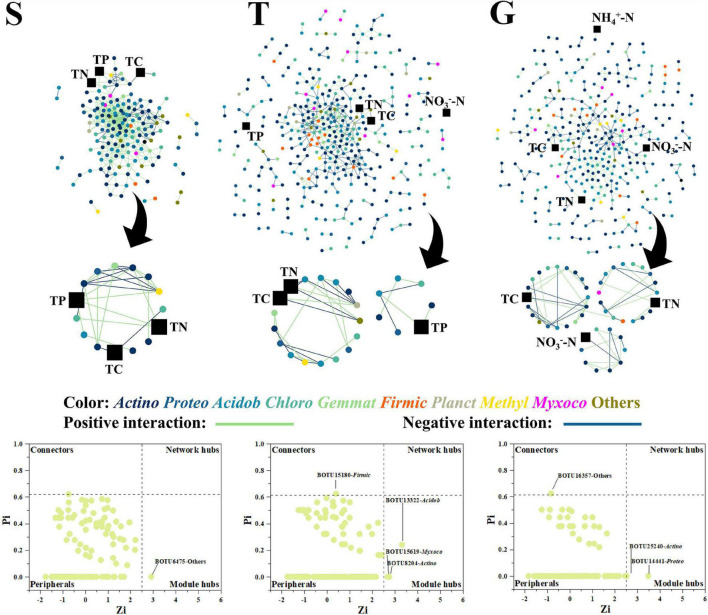
The bacterial network, with functional modules and key node composition. The phylogenetic identity (phylum level) of nodes is depicted by different colors; positive and negative interactions are depicted as green and blue lines, respectively. The environmental factors are depicted as black squares. The “*Actino*” is the abbreviation for “Actinobacteria” and similarly to other bacterial phylum.

While the bacterial network grew in size during the transition from shrubland (S) to grassland (G), it became less interactive, with the number of nodes increasing from 163 (S) to 375 (T) and 333 (G) but links decreasing from 607 (S) to 532 (T) and 411 (G). The corresponding average degrees (avgK) decreased from 7.45 (S) to 2.84 (T) and 2.47 (G). As the nodes were less clustered, the clustering coefficient (avgCC) decreased from 0.41 (S) to 0.19 (T) and 0.20 (G) (Figure 3 and Supplementary Table 2). Most importantly, TP gradually disappeared from the large central network modules, while ammonia and nitrate gradually appeared in the network modules (Figure 3). At the S stage, TP was present in a module with 15 (12 microbial) nodes and 26 links, while at the T stage, TP was in a module with only seven nodes and six links, and by the G stage, TP was not present in any of the modules, which indicated a shrinking TP-related functional module in the bacterial community network during shrub degradation. On the contrary, nitrate was not present in any module of network at the S stage but was in a peripheral module at T stage and a mid-sized module at G stage (10 nodes and 13 links) (Figure 3). Ammonia also appeared in a peripheral module at the G stage, which indicated a potential concentration on labile nitrogen formation of bacterial community during shrub degradation. However, the TC and TN were present in S, T, and G stages, which indicated relatively fixed bacterial TC, TN engaged functional modules during shrub degradation. The number of key network nodes increased from S to G. S had only 1 module hub (BOTU6475), while T and G had 3 and 2 module hubs, respectively, and each had 1 connector closely linking different modules, which indicated an increasing functional module interaction in bacterial network during shrub degradation.

**FIGURE 4:**
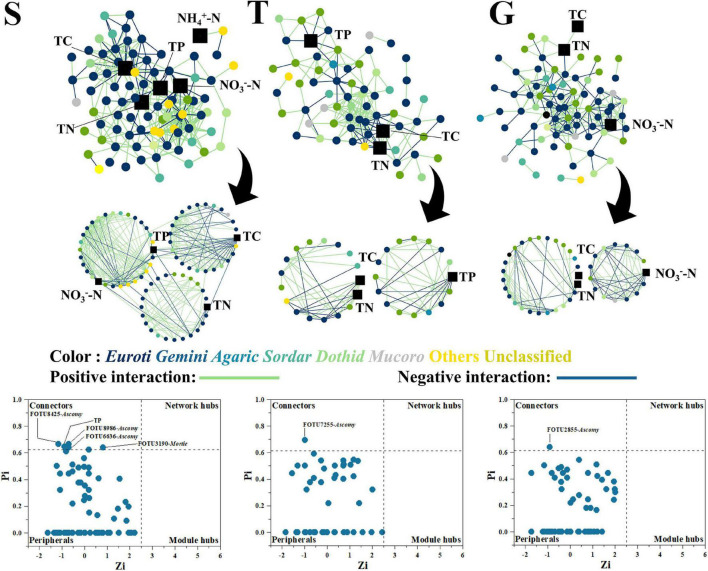
The fungal network, with functional modules and key node composition. The phylogenetic identity of nodes (class level) is depicted by different colors; network positive and negative interactions are depicted as green and blue lines, respectively. The environmental factors are depicted as black squares. The “*Euroti*” is the abbreviation for “Eurotiomycetes” and similarly to other fungal class.

On the contrary to the bacterial network, the fungal network became less member participated, shrinking from S (89 nodes) to T and G (68 and 79 nodes, respectively). S had 324 links, while T and G decreased to 188 and 222 links, respectively (Figure 4 and Supplementary Table 2). However, the fungal network also showed decreased interactivity as avgK changed from 7.28 (S) to 5.53 (T) and 5.62 (G), in a manner similar to the bacterial networks. The fungal network clustering state displayed no great variation among the three stages, as avgCC ranged between 0.39 (S) and 0.35 (T). Interestingly, TP also gradually disappeared in fungal network modules during shrub degradation, from a large central module in the S stage (30 nodes and 148 links) to a mid-sized module in the T stage (16 nodes and 35 links), and by G stage, it was not present in any modules (Figure 4). Moreover, TP acted as a connector at S, which indicated it had a strong effect on network organization at the S stage (Figure 4). Similar to bacterial networks, TC and TN were also present in S, T, and G stages, which indicated that the fungal network was also functionally constant about TC and TN during shrub degradation. However, different to bacterial network, key nodes of the fungal network had five connectors at S but only 1 connector each at T and G stages, displaying a decreasing trend, which indicated a decreasing functional module interaction in the fungal network during shrub degradation.

The results showed that the shrub site had a more member-participated fungal network and a less member-participated bacterial network than the other two stages. During the shrubland degradation by grass expansion, both bacterial and fungal network become less interactive and less focused on TP, with the bacterial network becoming more focused on nitrate and ammonia.

## Discussion

### TP, TN, and Shrub Degradation

In this study, shrubland degradation by grass expansion was accompanied by a decreasing TP and increasing TN, ammonia, and nitrate, which indicated an increase in relative P limitation (Figure 1C). This is unfavorable for shrub dominance as its higher P demand than grass needs to waste extra energy and material to uptake adequate P under P-limited condition (Yang et al., 2016; Carrillo et al., 2017). TP primarily decreased at the beginning of grass expansion (from S to T), while the further decrease during T to G was not as significant as during S to T, which indicated that the variation in TP was potentially the first step for shrub degradation. In the recent decade, Yalong dry valley tends to possess an increasing mean annual temperature, a drier wet season, and a wetter dry season (Sun et al., 2019). The drought-induced death of microbes stimulated the releasing of microbial organic P, and the following rewetting accelerated the leaching (Dinh et al., 2017; Brödlin et al., 2019). Since P leaching with precipitation is a major way for topsoil TP loss, the increasing frequency of dry-wet alteration might be a contributor to enhanced P limitation. In this research, TN, ammonia, and nitrate increased from S to G, which also enhanced relative P limitation indirectly. This is consistent to the conclusion from other ecosystems. Recent research found that nitrogen deposition released relative N limitation but increased P limitation and lead significant higher grass biomass increase rather than other species (Wang et al., 2021). Research on subalpine grassland also reported nitrogen deposition benefited grasses to a greater degree than shrub (Blanke et al., 2012). In this study, ammonia increased mostly during the later stage of expansion (from T to G) (Figures 1B,F,G), indicating that the final grass dominance over shrub was potentially linked to a more active nitrogen cycle. Compared with perennial shrubs, annual grass species tend to have faster growing rate (Uddin et al., 2020); fast-growing species had higher efficient in transporting water, in acquiring and using nutrients and in fixing carbon (Reich, 2014). Ammonia is released from the decomposition of organic nitrogen, a process conducted by microbes (Galloway et al., 2004). Moreover, ammonia is easily to be assimilated by fast-growing grass plants (Uddin et al., 2020), which could meet the higher nitrogen demand of grass species and enhance their dominance over shrubs (Galloway et al., 2004). Thus, our results support that enhanced relative P limitation, accompanied by the decreasing TP and increasing TN, potentially altered the relative competence of grass and shrub in this research.

### P Limitation Acts as a Main Driver of Microbial Community Composition Variation During Shrub Degradation

In this research, the SEM showed that P limitation exerted the highest total effect on both bacterial and fungal communities (Figures 2A,B), indicating that P limitation was the main driver of microbial community composition during grassland expansion of Tibetan dry valleys. This is possibly because the sampling site was mainly P-limited. The C:P in this research ranged from 111.77 ± 31.67 (S) to 176.99 ± 48.18 (G), according to global average soil microbial C:P (60:1); the microbial community in Tibetan dry valley tends to be more P-limited rather than C-limited as the field C:P offered less ratio P than microbes physiologically required (Cleveland and Liptzin, 2007). Similarly, global average soil microbial N:P was about 7:1, which was higher than 4.76 ± 0.91 (S) but lower than 10.55 ± 1.85 (T) and 12.11 ± 4.09 (G). These indicated that the microbial community faced a switch from less P-limited in S to a more P-limited state in T and G. As one of the basic nutrients to microorganisms, P was widely reported a driver to the variation on soil microbial community’s composition and function, especially under the P-limiting condition, including agricultural field (Su et al., 2015), grassland (Widdig et al., 2020a,b), and forest (Liu et al., 2012; Spohn et al., 2018). Thus, the microbial community composition in P-limited Tibetan dry valley was also mostly influenced by P limitation rather than pH and N resources.

However, the driver of microbial community composition during shrub-grass expansion varied in previous reports, and no congruent conclusions can be found. An Inner Mongolian grassland field investigation reported that TC and plant litter chemical composition played the most important role in regulating microbial composition (Zhou et al., 2017), and a subtropical marsh grassland study found that shrubs significantly altered microbial nitrogen cycle function, indicating nitrogen was the main driver of microbial community composition (Ho and Chambers, 2019). A separate report on Sayeret Shaked Park found that TC and TN were the main factors contributing to heterotrophic microbial composition change after the death of shrubs, while autotrophic microbial composition was controlled by soil moisture (Nejidat et al., 2016). This indicates that the detailed main driver of soil microbial community under shrub-grass change varies with climatic background, ecosystem types, microbial functional group concerned, etc. For example, a recent sub-continental investigation revealed that under dryer conditions, enhanced precipitation would release P limitation by accelerating rocky P mineralization. But under humid conditions, enhanced precipitation would increase P limitation by accelerating leaching (Li et al., 2022). Considering around 820 mm mean annual precipitation (relatively humid) in our sampling sites, P limitation tended to be amplified due to the higher precipitation in wet season. Moreover, P limitation would act as a main driver to microbial community’s composition in the climate change background in near future in Tibetan dry valley.

### The Change of Microbial Network Properties During Shrub Degradation

Our result showed that with the expansion of grass into shrub, both bacterial and fungal networks marginalized, then excluded TP from the central modules, and the bacterial network integrated nitrate and ammonia into mid-size (G) and peripheral modules (G), respectively (Figures 3, 4). A network module is a collection of microbial members that conduct highly linked functions (Deng and Zhou, 2015), and our results indicated a functional shift from a more P cycle-centered network to more nitrogen cycle-centered network. This was consistent with previous discussion that shrubs dominate under higher P content conditions, where the corresponding microbial community helps to turn over litter organic P into available soil phosphate (Yong et al., 2018; Gao et al., 2019, 2021), while grasses dominate under higher nitrogen conditions, where the corresponding microbial community accelerates nitrogen cycling and plant nitrogen assimilation (Galloway et al., 2004). Plant and soil microbial communities have been reported to select each other and mutually create a functional system that favors both sides (Veen et al., 2015). The plants were reported to be capable of shaping the soil community by altering the composition of their root exudates and litter falls to help obtain the nutrients they most require (Berendsen et al., 2012; Bruggen et al., 2019). In this study, shrubland soils had a more member-participated fungal network and a less member-participated bacterial network than transitional and grassland soils (Figures 3, 4), and the shrubland fungal network contained more key network nodes than the other sites, while the bacterial networks of transitional and grassland zones had more key network nodes than shrubland (Figures 3, 4), which indicated fungi played a greater role in S networks, while bacteria were more important in T and G networks. This is possibly due to the ecological function preference between bacterial members and fungal members. Compared with bacteria, fungi were reported to play a more important role in P cycling, as several members (mycorrhizal fungi) are strong P mineralizers (Smith et al., 2003). While bacteria play a more central roles in N cycling, as N fixation, nitrification and denitrification are mainly conducted by bacteria rather than fungi (Kuypers et al., 2018). Thus, higher P -demanding shrubs are prone to shape a more fungal member-participated network, while grasses shape a more member-participated bacterial network. Moreover, TP even acted as a connector in the S fungal network. Connectors are special nodes that link modules conduct different functions (Deng and Zhou, 2015). This indicated that the shrubland fungal community had several functions highly connected to TP. During further grass expansion, TP and the microbial community were not significantly changed, thus highlighting the disappearance of TP in the microbial network, a sign of functional change on the microbial network and a potential reason for further shrub degradation, as the microbial network changed to a state that could not support the P turnover rates to meet shrub demands. However, this requires further research on soil P component analysis and relative enzyme activities to confirm (Canfield et al., 2005; Chen et al., 2016).

## Conclusion

In this study, a grassland, a grass-shrub transitional field, and a shrubland from a Tibetan dry valley were chosen to investigate the microbial community variation change under a vegetation uplift expansion background. Consistent to our hypothesis, we found that during invasion by lower elevation grasses into higher elevation shrubs, P limitation was the main driver of both bacterial and fungal community composition. The soil network in shrubland was originally more P cycling-centered with a greater number of fungal members participating but gradually changed to a state that was less P cycling-centered and more bacterial members participating, which indicated that composition and network functional module change is a potential reason for shrub degradation. This research provides a microbial ecology mechanism to vegetation uplift expansion in Tibetan dry valleys and offers valuable information for dry valley ecosystem bioconservation and vegetation change response.

## Data Availability Statement

The datasets presented in this study can be found in online repositories. The names of the repository/repositories and accession number(s) can be found below: https://www.ncbi.nlm.nih.gov/, SAMN17245059.

## Author Contributions

HZ, AM, and GZ: design. HZ, JZ, XZ, and QZ: methodology. XC and HZ: software. HZ, JZ, and XQ: validation. HZ, JZ, and XC: formal analysis. HZ, JZ, QZ, and XC: investigation and resources. HZ: writing of the manuscript with help from AM, GL, and GZ. All authors involved in revising this manuscript, contributed intellectual input and assistance to this study, and manuscript preparation.

## Conflict of Interest

The authors declare that the research was conducted in the absence of any commercial or financial relationships that could be construed as a potential conflict of interest.

## Publisher’s Note

All claims expressed in this article are solely those of the authors and do not necessarily represent those of their affiliated organizations, or those of the publisher, the editors and the reviewers. Any product that may be evaluated in this article, or claim that may be made by its manufacturer, is not guaranteed or endorsed by the publisher.

## Footnotes

1
http://www.worldclim.org/

2
www.majorbio.com

3
http://ieg2.ou.edu/MENA/
