# Supplementary material for: Phosphorus Shapes Soil Microbial Community Composition and Network Properties During Grassland Expansion Into Shrubs in Tibetan Dry Valleys
Source: Front Plant Sci. 2022 Mar 23;13:848691. doi: 10.3389/fpls.2022.848691 (PMC8984195; doi:10.3389/fpls.2022.848691)
Supplement: Supplementary file 1 [file Data_Sheet_1.docx]

Supplementary Material


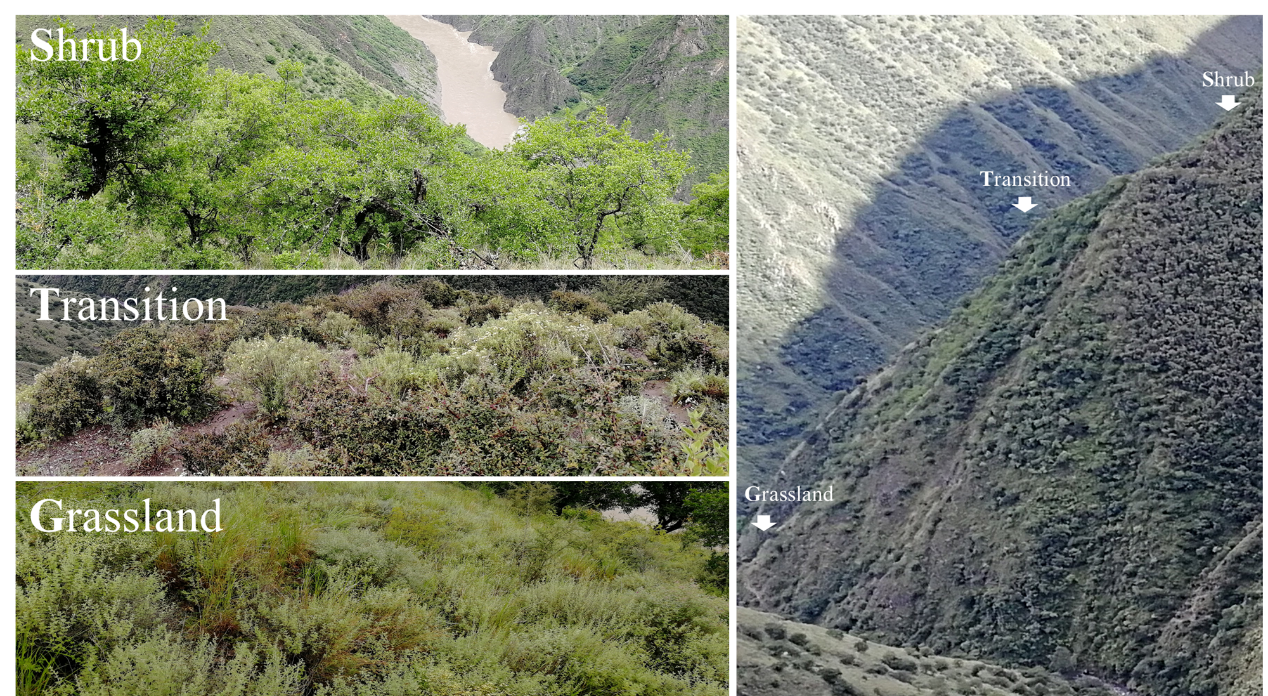


**Supplementary Figure 1.** The field photos of 3 sites and their location on the slope.


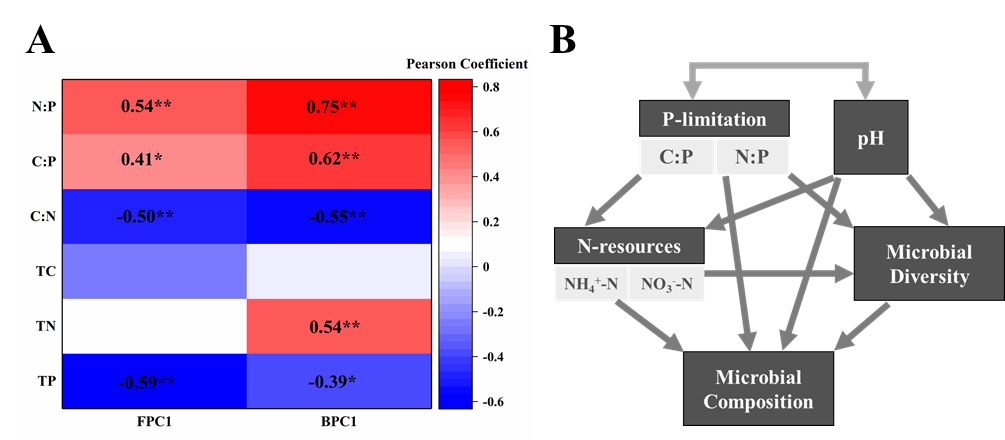


**Supplementary Figure 2.** The null model for structure equation modeling (SEM) resolving microbial community. The * and ** indicate significant correlated at *p* < 0.05 and *p* < 0.01 level, respectively. FPC1 and BPC1 indicate the first axis of PCoA results for fungal and bacterial community, respectively. TP, TN and TC are abbreviations for total phosphorus, total nitrogen and total carbon, respectively. The C:P, C:N and N:P are molar ratios.

The pearson correlation analysis showed that TP, rather than TN and TC, was significantly correlated to both FPC1 and BPC1, which indicated its importance in governing microbial community composition, thus we chose P related stoichiometry to compose SEM. C:P, N:P and C:N were all significantly correlated to both FPC1 and BPC1. Here N:P showed the highest coefficient over C:P and C:N, C:P had secondly high correlation coefficient to BPC1. Though the coefficient of C:P to FPC1 was lower than C:N, TP had highest (-0.59) coefficient than stoichiometry, which also indicated use P related stoichiometry is plausible for SEM.

In this model (B), C:P and N:P comprised the indicator of P-limitation, together with pH, they influenced the state of nitrogen resources (comprised by ammonia and nitrate), the diversity and composition of microbial community. And labile nitrogen resources, which the production and consumption is highly linked to environmental factors. The limitation on phosphorus would alter extracellular enzyme activity (like urease), pH would change the activity of nitrification and denitrification, and thus change the potential content of ammonia and nitrate. Labile nitrogen resources are also potentially influential to microbial composition by altering species pools (microbial diversity). Here, the effecting chain is P-limitation and pH to N-resources, then microbial diversity and finally microbial composition.

**Supplementary Table 1** NCBI project numbers for bacterial and fungal sequences

| Item names | Project number | Description |
| --- | --- | --- |
| DryvalleyBS | PRJNA788643 | Bacterial sequences of dry valley shrub |
| DryvalleyBT | PRJNA788647 | Bacterial sequences of dry valley shrub-grass transition |
| DryvalleyBG | PRJNA788654 | Bacterial sequences of dry valley grass |
| DryvalleyFS | PRJNA788662 | Fungal sequences of dry valley shrub |
| DryvalleyFT | PRJNA788673 | Fungal sequences of dry valley shrub-grass transition |
| DryvalleyFG | PRJNA788691 | Fungal sequences of dry valley grass |

**Supplementary Table 2** Topological properties of the empirical bacterial and fungal networks and their random network counterparts

|  | S bacterial network | | T bacterial network | | G bacterial network | | S fungal network | | T fungal network | | G fungal network | |
| --- | --- | --- | --- | --- | --- | --- | --- | --- | --- | --- | --- | --- |
| Network | Em | Ran | Em | Ran | Em | Ran | Em | Ran | Em | Ran | Em | Ran |
| Total nodes | 163 |  | 375 |  | 333 |  | 89 |  | 68 |  | 79 |  |
| Total links | 607 |  | 532 |  | 411 |  | 324 |  | 188 |  | 222 |  |
| Average clustering coefficient (avgCC) | 0.41 | 0.103±0.010* | 0.191 | 0.009±0.004* | 0.198 | 0.007±0.004* | 0.385 | 0.145±0.018* | 0.345 | 0.160±0.023* | 0.363 | 0.111±0.016* |
| Average path distance (GD) | 4.034 | 2.817±0.039* | 5.747 | 5.008±0.090* | 8.306 | 5.737±0.137* | 3.12 | 2.549±0.035* | 3.623 | 2.662±0.054* | 3.352 | 2.756±0.063* |
| Geodesic efficiency (E) | 0.322 | 0.399±0.004* | 0.224 | 0.226±0.003 | 0.169 | 0.202±0.004* | 0.395 | 0.447±0.004* | 0.372 | 0.434±0.006* | 0.374 | 0.420±0.007* |
| Harmonic geodesic distance (HD) | 3.109 | 2.509±0.026* | 4.467 | 4.424±0.066* | 5.908 | 4.957±0.097* | 2.529 | 2.237±0.020* | 2.689 | 2.304±0.032* | 2.671 | 2.383±0.039* |
| Centralization of betweenness (CB) | 0.116 | 0.067±0.009* | 0.052 | 0.095±0.017* | 0.11 | 0.096±0.017 | 0.106 | 0.090±0.015* | 0.139 | 0.136±0.021 | 0.143 | 0.109±0.019* |
| Centralization of stress centrality (CS) | 2.505 | 0.319±0.036* | 0.256 | 0.234±0.044 | 0.351 | 0.183±0.032* | 0.686 | 0.353±0.043* | 3.428 | 0.432±0.055* | 0.633 | 0.361±0.052* |
| Centralization of eigenvector centrality (CE) | 0.227 | 0.178±0.012* | 0.307 | 0.270±0.046 | 0.323 | 0.248±0.038* | 0.251 | 0.190±0.013* | 0.262 | 0.232±0.014* | 0.256 | 0.205±0.016* |
| Transitivity (Trans) | 0.484 | 0.137±0.008* | 0.297 | 0.016±0.005* | 0.307 | 0.012±0.007* | 0.456 | 0.165±0.012* | 0.498 | 0.196±0.016* | 0.426 | 0.143±0.013* |
| Connectedness (Con) | 0.802 | 0.967±0.027* | 0.303 | 0.823±0.038* | 0.385 | 0.770±0.041* | 0.891 | 0.992±0.019* | 0.806 | 0.975±0.033* | 0.854 | 0.966±0.038* |
| Efficiency | 0.95 | 0.958±0.001* | 0.982 | 0.994±0.000* | 0.987 | 0.994±0.000* | 0.918 | 0.927±0.002* | 0.913 | 0.929±0.003* | 0.929 | 0.937±0.003* |
| Modularity(fast_greedy) | 0.496 | 0.294±0.007* | 0.792 | 0.645±0.007* | 0.859 | 0.712±0.007* | 0.475 | 0.278±0.009* | 0.466 | 0.317±0.013* | 0.525 | 0.328±0.011* |

Notes: “Em” indicates empirical networks, “Ran” indicate random networks. * indicates significant differences between empirical network and random network parameters of the same group (*p* < 0.05). For “Ran”, the standard deviation was attached to average. S, T and G in headline indicate shrub stage, transition stage and grassland stage, respectively.
